# Constructing and validating a prognostic prediction nomogram model for hepatocellular carcinoma patients following high-intensity focused ultrasound treatment

**DOI:** 10.3389/fonc.2026.1789920

**Published:** 2026-05-14

**Authors:** Hanyu Huang, Fan Yang, Pengcheng Liu, Kun Zhou, Wenzhi Chen

**Affiliations:** 1 State Key Laboratory of Ultrasound in Medicine and Engineering, College of Biomedical Engineering, Chongqing Medical University, Chongqing, China; 2 Department of Oncology, Suining Central Hospital, Suining, China; 3 Clinical Center for Tumor Therapy, 2nd Affiliated Hospital, Chongqing Medical University, Chongqing, China

**Keywords:** hepatocellular carcinoma, HIFU, nomogram, overall survival, prognosis model

## Abstract

**Objective:**

To develop and validate a nomogram model based on biological information and conventional imaging indicators for predicting overall survival (OS) in patients with hepatocellular carcinoma (HCC) following high-intensity focused ultrasound (HIFU) treatment.

**Methods:**

This retrospective study included 407 patients with HCC who received HIFU treatment at the Second Affiliated Hospital of Chongqing Medical University between January 1, 2013, and June 30, 2024. Patients were randomly divided into a training cohort (n = 244) and a validation cohort (n = 163) at a ratio of 6:4. In the training cohort, univariate and multivariate Cox regression analyses were performed to identify independent predictors of OS. A nomogram was subsequently constructed to predict 1, 3, and 5-year survival rates. The predictive performance of the model was evaluated by assessing the concordance index (C−index), area under the receiver operating characteristic curve (AUC), calibration curves, and decision curve analysis (DCA). Kaplan-Meier survival curves were plotted to compare survival between high−risk and low−risk groups stratified by the nomogram, thereby validating the model’s ability for clinical risk stratification.

**Results:**

Univariate Cox regression analysis identified 15 factors significantly associated with OS. Multivariate Cox analysis further determined that lymphocyte count, maximum tumor diameter, alpha fetoprotein level, number of tumor lesions, and portal vein invasion were independent risk factors. In the training cohort, the model exhibited a C index of 0.783. The AUCs for 1, 3, and 5-year survival were 0.814, 0.895, and 0.825, respectively. Calibration curves for 1, 3, and 5-year survival showed close agreement between the nomogram-predicted probabilities (0.835, 0.587, 0.40) and the observed survival rates (0.843, 0.586, 0.551), indicating excellent consistency. In the validation cohort, the C-index was 0.701, and the AUCs were 0.778, 0.679, and 0.754, respectively. The calibration curves also demonstrated good agreement between predicted and observed survival rates, reflecting acceptable consistency. In comparison, the 8th edition AJCC staging system yielded C indices below 0.68 in both cohorts, and its calibration curves showed suboptimal fit.

**Conclusion:**

The nomogram model developed in this study can effectively predict the OS rates in HCC patients following HIFU treatment, potentially improving therapeutic strategies and promoting personalized treatment approaches.

## 1 Introduction

Hepatocellular carcinoma (HCC) is the most common malignant liver tumor, with its incidence showing an upward trend. According to the latest global cancer statistics, HCC ranks sixth in annual new cases and third in deaths among malignant tumors, imposing a heavy burden on healthcare systems (1). Surgical resection remains the primary treatment for HCC, but due to its insidious onset, only 15% of patients can benefit from surgical intervention (2). For patients unsuitable for surgery or those with recurrent HCC, local treatment modalities such as transcatheter arterial chemoembolization (TACE), high-intensity focused ultrasound (HIFU), radiofrequency ablation (RFA), percutaneous ethanol injection (PEI), cryoablation, microwave ablation (MWA), and stereotactic body radiation therapy (SBRT) hold significant clinical value and importance (3–5).

HIFU offers a novel therapeutic option for HCC patients with tumors adjacent to critical structures or limited hepatic functional reserve, owing to its unique non-invasive and radiation-free advantages (6). Through the precise external focusing of low-energy ultrasonic waves on the target tumor, HIFU instantly generates temperatures of 65-100°C at the lesion site by means of thermal, cavitation, and mechanical effects. This results in coagulative necrosis of the tumor while preserving surrounding normal tissues, thereby achieving the effect of a non-invasive surgery (7). Multiple studies have confirmed that HIFU, either as a monotherapy or in combination with other modalities such as TACE, can achieve favorable local control and effectively prolong survival in HCC patients at specific stages (8, 9). However, long-term outcomes following HIFU treatment vary significantly among individuals, with some patients still facing risks of tumor recurrence or progression (10–13). Therefore, accurately assessing and predicting individualized prognosis prior to treatment holds crucial guiding value for formulating optimal therapeutic strategies and enabling precision management of patients.

Currently, several staging systems such as the Tumor-Node-Metastasis (TNM) and Barcelona Clinic Liver Cancer (BCLC) staging systems (14, 15), have been employed for prognostic prediction in HCC. However, the prognosis of HCC is not solely dependent on tumor stage. Numerous studies have shown that many risk factors, including gender, age, treatment modality, and hepatitis virus infection, also significantly influence the survival outcomes of HCC patients (16, 17). Consequently, traditional staging systems alone are insufficient for accurately assessing patient prognosis. In recent years, prognostic prediction models, particularly visual models presented in the form of nomograms, have demonstrated considerable potential in the field of oncology outcome evaluation due to their ability to integrate multiple factors and perform individualized probability calculations (17, 18). Although studies have developed prediction models applicable to patients after liver resection, TACE, RFA, and MWA (19–22), there is currently a lack of a dedicated prognostic tool for predicting overall survival (OS) in HCC patients following HIFU treatment—one that comprehensively incorporates multidimensional information such as tumor burden, biological characteristics, and the host immune microenvironment.

Considering the preceding circumstances, this study aims to construct a visual nomogram prediction model by retrospectively analyzing the clinical data of HCC patients treated with HIFU. Independent prognostic factors will be identified using univariate and multivariate Cox regression analyses. The accuracy of the model in distinguishing between patients with different survival outcomes will be quantified using the Concordance index (C-index) and the area under the receiver operating characteristic curve (AUC). Calibration curves will be employed to assess the consistency between the model’s predicted probabilities and the observed survival rates, thereby ensuring the reliability of its outputs. Decision curve analysis (DCA) will be used to evaluate the net clinical benefit provided by the model across various risk thresholds. Kaplan-Meier survival curves were plotted to compare survival between high−risk and low−risk groups stratified by the nomogram, thereby validating the model’s ability for clinical risk stratification (23, 24). Finally, the predictive performance metrics of our model will be compared with those of the 8th edition American Joint Committee on Cancer (AJCC) TNM staging system. We anticipate that this model will offer a more precise and practical quantitative tool for prognostic assessment and individualized treatment decision-making in HCC patients undergoing HIFU therapy.

## 2 Materials and methods

### 2.1 Patients

This study enrolled patients with HCC who underwent HIFU therapy at the Second Affiliated Hospital of Chongqing Medical University between January 1, 2013, and June 30, 2024. Inclusion criteria were as follows: (1) age ≥ 18; (2) HCC confirmed by histopathology or imaging; (3) complete clinical records and follow-up data; and (4) availability of high-quality computed tomography (CT) or magnetic resonance imaging (MRI) images before and after treatment. Exclusion criteria included: (1) history of radiofrequency ablation within 6 months before HIFU; (2) severe motion or respiratory artifacts on MRI that hampered evaluation; (3) prior liver resection or transplantation within 6 months before treatment; (4) loss to follow-up after discharge; (5) death within 90 days after HIFU; and (6) cases without definitive imaging diagnosis and lacking pathological confirmation.

Given the total sample size and number of events, and to balance statistical power (Events Per Variable, EPV > 10) required for model construction with the robustness of validation results, enrolled patients were randomly divided into training and validation cohorts at a ratio of 6:4.

This study was approved by the Institutional Ethics Review Committee of The Second Affiliated Hospital of Chongqing Medical University (Ethics Approval No.: (2024) 94) and was conducted in accordance with the ethical principles of the World Medical Association Declaration of Helsinki. Due to the retrospective nature of the study, the ethics committee waived the requirement for informed consent.

### 2.2 HIFU therapy

The HIFU procedure was performed using the focused ultrasound tumor therapeutic system (Model-JC) manufactured by Chongqing Haifu Technology Co., Ltd. An imaging ultrasound probe (frequency range 3.5–5.0 MHz) provided real-time ultrasound imaging for localization of the target lesion and continuous monitoring throughout the HIFU treatment session.

Key parameters of the therapeutic transducer, including its diameter (100–300 mm), focal length (100–250 mm), and operating frequency (0.5–2 MHz), constituted the core elements enabling the conversion of electrical energy into ultrasonic energy and the formation of an effective focused ultrasound field. The focal region was ellipsoidal in shape, with dimensions of 3 mm x 8 mm.

The standard treatment position was right lateral decubitus or prone position. Before the procedure, the operator used real-time ultrasound imaging combined with three-dimensional reconstructed images to precisely delineate the tumor location, size, morphology, and its relationship with adjacent tissues, thereby ensuring the safety of the acoustic pathway.

Preoperative HIFU treatment planning encompassed both the intrahepatic tumor lesions and portal vein tumor thrombus (PVTT). For some patients, factors such as large intrahepatic tumor diameter (> 15 cm), proximity of the lesion margin to hollow organs (e.g., stomach, intestine, gallbladder; distance < 0.5 mm), or severe liver cirrhosis with liver function remaining at Child–Pugh class B despite medical therapy, might necessitate a staged treatment approach or ablation of only a portion of the tumor.

In the present cohort, the acoustic power applied during HIFU ranged from 100 to 400 W. The total treatment time per session ranged from 85 to 8000 s, reflecting the heterogeneity of tumor size and complexity.

### 2.3 Data collection

The following data were collected for enrolled patients via the Hospital Information System (HIS): age, sex, preoperative laboratory parameters: (platelet count (PLT), white blood cell count (WBC), neutrophil count (NEU), lymphocyte count (LYMPH), monocyte count (MON), albumin (ALB), total bilirubin (TBIL), aspartate aminotransferase (AST), alanine aminotransferase (ALT), alpha−fetoprotein level (AFP), prothrombin time (PT), viral hepatitis infection and virus type, ascites, hepatic encephalopathy (HE), hypertension, diabetes, cirrhosis, jaundice), preoperative liver function scores: (Child–Pugh grade, Albumin-Bilirubin (ALBI) grade), TNM stage, Imaging characteristics: (MRI signal characteristics, involvement of hepatic artery/vein/portal vein (PV), number of HCC lesions, maximum diameter, location, intralesional necrosis/hemorrhage/fat, perilesional enhancement, metastasis), HIFU Treatment Parameters: (treatment duration, power, number of treated lesions, maximum diameter), whether combined with TACE or Transarterial embolization (TAE), systemic therapy and regimen (chemotherapy, immunotherapy, targeted therapy, or combined targeted−immunotherapy), OS.

Of note, the present study aimed to develop a pre-operative prognostic prediction model, therefore, post-treatment variables such as immediate HIFU response and subsequent adjuvant therapies were not included in the primary model.

### 2.4 Follow-up

All enrolled patients were regularly followed. During the first 2 years, follow−up was conducted every 3 months, and thereafter every 3–6 months until death or withdrawal from the study. Patients who did not attend scheduled hospital visits were contacted by telephone to obtain information regarding treatment status and general condition. The final follow−up date was June 2025. OS was defined as the interval from the date of HIFU treatment to the date of death or the last follow−up.

### 2.5 Statistical analysis

Continuous variables were described according to their distribution characteristics. Continuous variables following a normal distribution were expressed as mean ± standard deviation (SD), while non-normally distributed continuous variables were presented as median and interquartile range (IQR). Comparisons of continuous variables were performed using the Mann-Whitney U test or Student’s t-test. Categorical variables were expressed as frequencies (percentages) and compared using the Chi−square (χ²) test or Fisher’s exact test. Optimal cut−off values for continuous variables were determined using X−tile software (version 3.6.1; https://x-tile.software.informer.com/) and subsequently dichotomized. Variables with p < 0.05 in univariate analysis were entered into multivariate Cox regression analysis. The proportional hazards assumption was assessed using Schoenfeld residual testing. Nomogram models were developed and validated employing R packages including ‘rms’, ‘survival’, ‘timeROC’, and ‘glmnet’. Model performance was evaluated using the C−index, AUC, and calibration curves. The clinical utility was further assessed using DCA and Kaplan-Meier curves with the log-rank test. A two−tailed p value < 0.05 was considered statistically significant.

All statistical analyses were performed using SPSS software (version 27.0; IBM, Armonk, NY, USA) and R software (version 3.5.3; https://www.r-project.org/).

## 3 Results

### 3.1 Clinical characteristics

**Figure 1:**
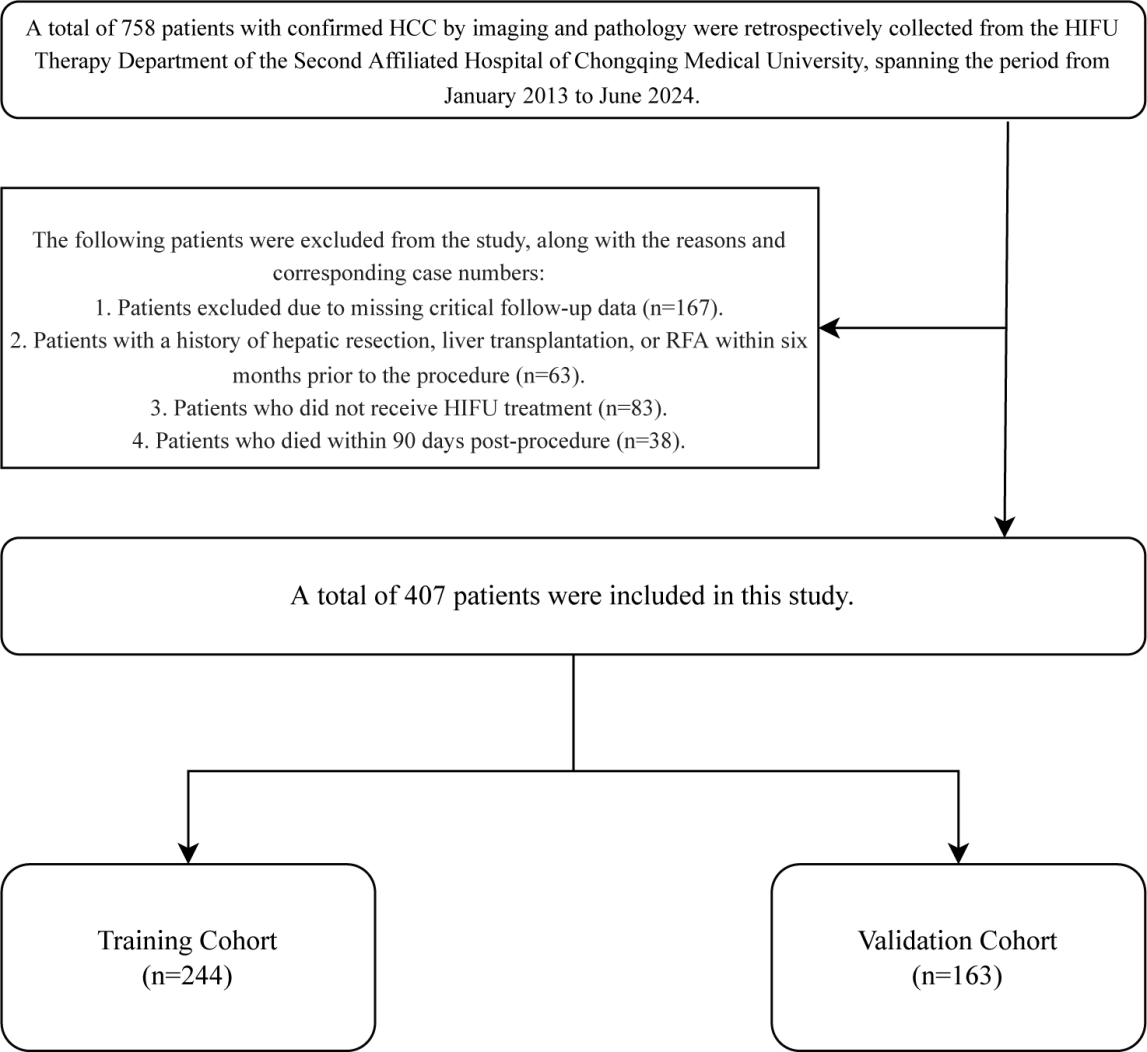
Patient selection of the study.

A total of 758 patients were initially screened for the study. Based on the predefined inclusion and exclusion criteria, 351 patients were excluded. The main reasons for exclusion were: 167 due to lack of essential follow-up data, 63 with a history of liver resection, liver transplantation, or RFA within six months before HIFU treatment, 83 who did not undergo HIFU therapy, and 38 who died within 90 days postoperatively. Consequently, 407 eligible patients were ultimately enrolled and randomly allocated into a training cohort and a validation cohort at a 6:4 ratio, as illustrated in **Figure 1**.

The cohort had a mean age of 55.05 ± 11.74 years, with a predominance of male patients (87.5%) over female patients (12.5%). Over a mean follow-up period of 32.88 months, 108 death events (26.5%) were observed. All deaths were attributed to HCC progression or its complications. The OS for the entire cohort was 65 months (95% confidence interval (CI): 35.822-94.178).

In the training cohort (n = 244), the median OS was 65 months (95% CI: 29.875–100.125), which was comparable to the median OS of 65 months (95% CI: 24.974–105.026) observed in the validation cohort (n = 163), with no statistically significant difference between the two cohorts (p = 0.669). The mean follow-up time was 34.14 months (95% CI: 28.736–39.536) in the training cohort and 30.934 months (95% CI: 25.611–36.257) in the validation cohort, indicating no significant difference (p = 0.839). At the last follow-up, 67 patients (27.5%) in the training cohort and 41 patients (25%) in the validation cohort had died; this difference was not statistically significant (p = 0.606). Overall, the baseline clinical characteristics between the training and validation cohorts were well-balanced, with no significant differences (p > 0.05; **Table 1**).

**Table 1:** Comparison of baseline clinical and pathological characteristics between training and validation cohorts.

Variable	Training cohort (N = 244)	Validation cohort (N = 163)	P value
Gender (male/female)	214/30	142/21	0.861
Age	54.71 ± 11.85	55.56 ± 11.58	0.477
Tumor max diameter (cm)			0.407
≤ 5	161	101	
> 5	83	62	
PLT IQR (10^9^/L)	112.00 (73.75, 171.00)	122.00 (75.50, 180.00)	0.341
WBC IQR (10^9^/L)	4.73 (3.60, 6.27)	4.55 (2.55)	0.149
MON IQR (10^9^/L)	0.37 (0.27, 0.54)	0.37 (0.25, 0.50)	0.769
NEU IQR (10^9^/L)	2.79 (2.15, 4.03)	2.74 (1.92, 3.82)	0.193
LYMPH IQR (10^9^/L)	1.98 (1.07, 4.52)	2.64 (1.17, 4.93)	0.295
ALB (g/L)	38.78 ± 5.85	38.10 ± 5.69	0.249
ALT IQR (IU/L)	31.00 (23.00, 50.00)	35.00 (23.00, 49.50)	0.638
AST IQR (IU/L)	41.50 (29.00, 65.00)	41.00 (29.00, 66.00)	0.838
TBIL IQR (umol/L)	14.30 (10.30, 21.45)	15.40 (10.40, 23.55)	0.464
AFP IQR (ng/ml)	99.97 (10.58, 1210.00)	90.87 (6.78, 1161.50)	0.335
PT IQR (s)	13.90 (13.20, 14.90)	13.90 (13.20, 14.75)	0.839
Hepatitis B (Yes/No)	212/32	135/28	0.257
Hepatitis C (Yes/No)	6/238	4/159	1.000
Jaundice (Yes/No)	15/229	14/149	0.348
Cirrhosis of the liver (Yes/No)	164/80	104/59	0.477
Ascites (Yes/No)	70/174	49/114	0.765
HE (Yes/No)	9/235	6/157	0.997
Hypertension (Yes/No)	44/200	39/124	0.148
Diabetes (Yes/No)	33/211	24/139	0.733
Invasion of hepatic artery (Yes/No)	3/241	1/162	0.917
Invasion of PV (Yes/No)	80/164	45/118	0.267
Invasion of hepatic vein (Yes/No)	40/204	22/141	0.426
Clear boundaries (Yes/No)	73/171	46/117	0.712
Tumor capsule intact (Yes/No)	15/229	12/151	0.630
Tumor amounts			0.790
Single	109	75	
Multi	135	88	
Tumor location			0.633
Single-leaf	153	106	
Double-leaf	91	57	
Distant metastasis (Yes/No)	91/153	48/115	0.102
Lymph node metastasis (Yes/No)	34/210	16/147	0.215
TNM stage			0.257
I	43	40	
II	57	42	
IIIA	15	8	
IIIB	19	17	
IVA	19	8	
IVB	91	48	
Treatment method			0.921
HIFU	41	28	
HIFU+TAE/TACE	203	135	
Therapeutic power IQR (W)	400.00 (367.75, 400.00)	400.00 (363.50, 400.00)	0.891
Therapeutic time IQR (s)	851.00 (459.25, 1570.25)	801.00 (473.50, 1510.00)	0.615
Therapeutic diameter IQR (cm)	5.00 (4.00, 8.00)	5.00 (4.00, 8.00)	0.423
Therapeutic amounts			0.975
< 3	171	114	
≥ 3	73	49	
Child-Pugh			0.875
A	190	128	
B&C	54	35	
Artery Phase enhancement			0.697
Significantly enhanced	234	155	
Lowly enhanced	10	8	
PV phase enhancement			0.131
Significantly enhanced	177	129	
Lowly enhanced	67	34	
Intratumoral fat (Yes/No)	4/240	4/159	0.829
Intratumoral hemorrhage (Yes/No)	9/235	2/161	0.235
Intratumoral necrosis (Yes/No)	73/171	43/120	0.439
Peritumoral enhancement (Yes/No)	31/213	12/151	0.086
Systemic therapy (Yes/No)	141/103	86/77	0.317
Chemotherapy	5	1	
Immunotherapy	18	18	
Targeted therapy	18	7	
Targeted-immunotherapy	102	60	
ALBI score			0.701
1	117	75	
2&3	127	88	
Recurrence (Yes/No)	125/119	79/84	0.585
History of hepatectomy (Yes/No)	45/199	23/140	0.251
History of RFA (Yes/No)	26/218	12/151	0.263
Etiology of HCC			0.479
Viral	214	139	
Non-viral	30	24	

### 3.2 Risk factors in the training cohort

Univariate and multivariate Cox regression analyses were conducted in the training cohort. For continuous variables with established clinical standards or broad consensus (e.g., tumor size, dichotomized at 5 cm according to TNM staging criteria), those criteria were directly applied for dichotomization. For continuous variables without clear clinical cutoffs, the optimal cutoff was determined based on its association with survival outcomes using X-tile software.

Univariate analysis of clinical risk factors (**Table 2**) revealed that preoperative AST, TBIL, WBC, LYMPH, MON, PLT, AFP, jaundice, maximum tumor diameter, number of tumor lesions, Child–Pugh grade, radiologic lymph node metastasis, hepatic vein invasion, portal vein invasion, and tumor border were associated with OS (p < 0.05). Multivariate Cox regression analysis identified five independent risk factors for poor prognosis: preoperative LYMPH (hazard ratios (HR) = 0.339, 95% CI: 0.194–0.590, p < 0.001), maximum tumor diameter (HR = 2.563, 95% CI: 1.490–4.410, p < 0.001), AFP (HR = 2.231, 95% CI: 1.325–3.758, p = 0.003), number of tumor lesions (HR = 2.941, 95% CI: 1.701–5.087, p < 0.001), and portal vein invasion (HR = 2.430, 95% CI: 1.362–4.334, p = 0.003) (**Table 2**). A risk score was calculated using the following formula: Risk score = -1.083 × Preoperative LYMPH (1 (≤ 0.9×10^9^/L) or 0 (> 0.9×10^9^/L)) + 0.803 × AFP (0 (≤ 99.5 ng/mL) or 1 (> 99.5 ng/mL)) + 1.079 × Number of tumor lesions (0 (single) or 1 (multiple)) + 0.888 × Portal vein invasion (0 (no) or 1 (yes)) + 0.941 × Maximum tumor diameter (0 (≤ 5 cm) or 1 (> 5 cm)).

**Table 2:** Univariate and multivariate cox regression analyses of preoperative clinical characteristics in the training cohort (n=244).

Variable	Univariate analysis		Multivariate analysis	
	HR (95% CI)	P value	HR (95% CI)	P value
Age	0.984 (0.962–1.006)	0.148		
Gender				
Male vs. Female	1.398 (0.665–2.939)	0.377		
Tumor max diameter				
> 5cm vs.≤ 5cm	2.659 (1.612–4.386)	< 0.001^*^	2.563 (1.490–4.410)	< 0.001^*^
Tumor Amounts				
Multi vs. Single	2.534 (1.495–4.293)	< 0.001^*^	2.941 (1.701–5.087)	< 0.001^*^
PLT (10^9^/L)				
> 47 vs. ≤ 47	0.480 (0.260–0.887)	0.019^*^	0.588 (0.279–1.238)	0.162
WBC (10^9^/L)				
> 2.6 vs. ≤ 2.6	0.500 (0.261–0.958)	0.037^*^	0.710 (0.315–1.610)	0.410
NEU (10^9^/L)				
> 5.6 vs. ≤ 5.6	1.740 (0.790–3.833)	0.169		
LYMPH (10^9^/L)				
> 0.9 vs. ≤ 0.9	0.420 (0.246–0.719)	0.002^*^	0.338 (0.194–0.590)	< 0.001^*^
MON (10^9^/L)				
> 0.8 vs. ≤ 0.8	2.227 (1.050–4.722)	0.037^*^	1.967 (0.853–4.541)	0.113
ALB (g/L)				
> 30.9 vs. ≤ 30.9	0.966 (0.928–1.006)	0.096		
TBIL (umol/L)				
> 32.1 vs. ≤ 32.1	2.969 (1.608–5.483)	< 0.001^*^	1.731 (0.721–4.156)	0.219
ALT (IU/L)				
> 34 vs. ≤ 34	0.719 (0.438–1.181)	0.193		
AST (IU/L)				
> 92vs. ≤ 92	3.750 (2.018–6.969)	< 0.001^*^	1.652 (0.792–3.452)	0.181
AFP (ng/ml)				
> 99.5 vs. ≤ 99.5	2.657 (1.607–4.394)	< 0.001^*^	2.231 (1.325–3.758)	0.003^*^
PT (s)				
> 15 vs. ≤ 15	1.477 (0.871–2.506)	0.148		
Jaundice				
Yes vs. No	3.060 (1.386–6.756)	0.006^*^	1.313 (0.475–3.628)	0.599
Hepatitis B				
Yes vs. No	1.305 (0.561–3.033)	0.536		
Cirrhosis of the liver				
Yes vs. No	0.823 (0.489–1.386)	0.464		
Ascites				
Yes vs. No	1.620 (0.980–2.677)	0.060		
HE				
Yes vs. No	2.216 (0.888–5.528)	0.088		
Hypertension				
Yes vs. No	0.608 (0.290–1.276)	0.188		
Diabetes				
Yes vs. No	0.794 (0.379–1.665)	0.542		
Child-Pugh				
B/C vs. A	2.103 (1.257–3.516)	0.005^*^	1.360 (0.664-2.785)	0.400
ALBI				
2/3 vs. 1	1.227 (0.750–2.006)	0.415		
Invasion of hepatic artery				
Yes vs. No	2.361 (0.325–17.136)	0.396		
Invasion of PV				
Yes vs. No	2.442 (1.439–4.143)	< 0.001^*^	2.430 (1.362–4.334)	0.003^*^
Invasion of hepatic vein				
Yes vs. No	2.408 (1.229–4.720)	0.010^*^	0.957 (0.417–2.195)	0.917
Distant metastasis				
Yes vs. No	1.546 (0.951–2.512)	0.079		
Lymph node metastasis				
Yes vs. No	4.354 (2.339–8.106)	< 0.001^*^	2.020 (0.934–4.372)	0.074
Intra-tumoral condition				
Hemorrhage				
Yes vs. No	2.553 (0.924–7.050)	0.071		
Necrosis				
Yes vs. No	1.129 (0.667–1.911)	0.652		
Fat				
Yes vs. No	0.473 (0.065–3.431)	0.459		
Peritumoral enhancement				
Yes vs. No	1.744 (0.910–3.340)	0.094		
Tumor capsule				
Yes vs. No	0.452 (0.141–1.446)	0.181		
Tumor margin				
Smooth vs. Rough	0.576 (0.332–0.998)	0.049^*^	1.134 (0.605–2.128)	0.695
Artery phase enhancement				
Low/Medium vs. High	0.819 (0.256–2.615)	0.736		
PV phase enhancement				
Low/Medium vs. High	0.986 (0.579–1.684)	0.963		
Recurrence				
Yes vs. No	1.375 (0.843–2.242)	0.202		
Systemic therapy				
Yes vs. No	1.544 (0.936–2.549)	0.089		
Chemotherapy	2.720 (0.986–7.504)	0.053		
Immunotherapy	0.494 (0.155–1.576)	0.234		
Targeted therapy	2.021 (0.919–4.448)	0.08		
Targeted-immunotherapy	1.290 (0.790–2.106)	0.31		
History of hepatectomy				
Yes vs. No	1.262 (0.699–2.279)	0.440		
History of RFA				
Yes vs. No	0.738 (0.318–1.709)	0.478		
Etiology of HCC				
Viral vs. Non-viral	0.890 (0.405–1.953)	0.771		

### 3.3 Model development and validation

Based on the five independent risk factors identified from the multivariate regression analysis—preoperative LYMPH, maximum tumor diameter, AFP, number of tumor lesions, and portal vein invasion—a clinical prediction model was successfully developed to estimate OS in HCC patients after HIFU treatment. This model was visualized as a risk nomogram.

**Figure 2:**
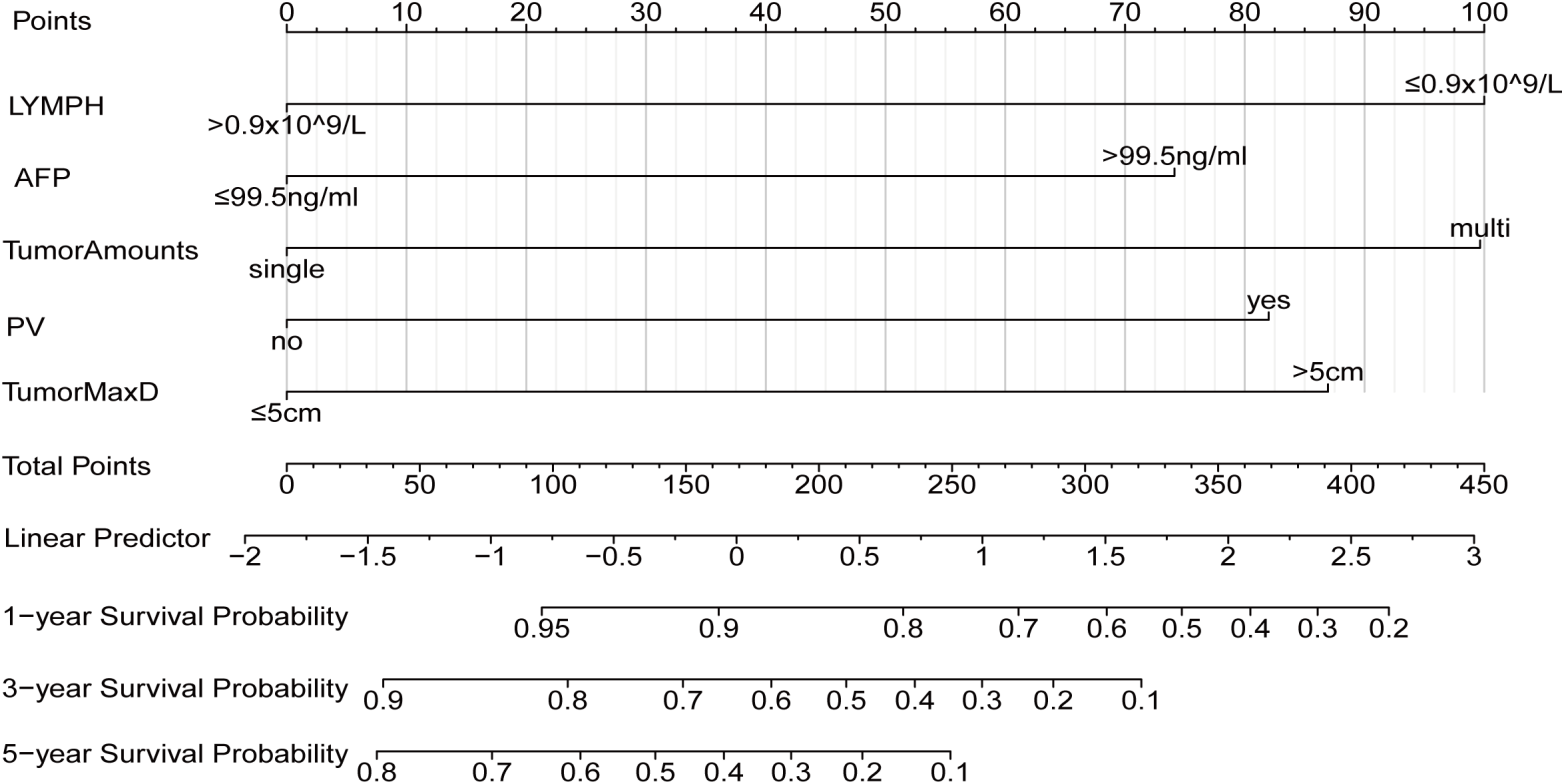
Nomogram based on independent clinical risk predictors. LYMPH: Lymphocyte count; AFP: Alpha-fetoprotein; PV: Portal vein; TumorMaxD: Maximum tumor diameter. For PV, “no” indicates no invasion, while “yes” indicates invasion.

As shown in **Figure 2**, the nomogram allows estimation of the predicted 5 year survival probability for individual patients. For example, in a real HCC patient who underwent HIFU treatment, with a preoperative LYMPH of 3.58×10^9^/L (>0.9×10^9^/L, assigned 0 points on the nomogram), a maximum tumor diameter of 6 cm (> 5 cm, 86 points), AFP of 416 ng/mL (> 99.5 ng/mL, 74 points), a single tumor lesion (0 points), and no portal vein invasion (0 points), the total score sums to 160 points. Based on this score, the predicted probabilities of survival after surgery were 91% at 1 year, 68% at 3 years, and 45% at 5 years.

**Figure 3:**
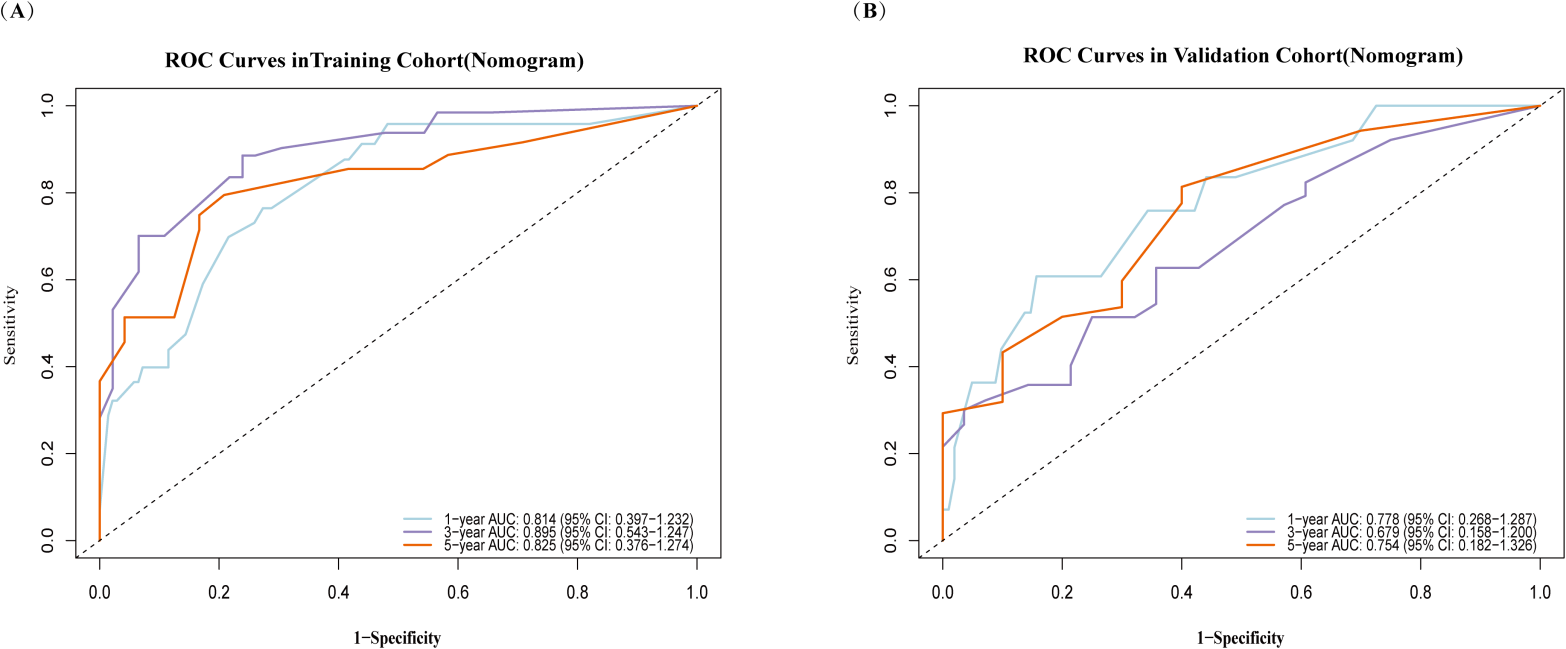
Time-dependent ROC curves for predicting postoperative survival rates in HCC following HIFU, based on the nomogram model. **(A)** Training cohort; **(B)** validation cohort. AUC for predicting 1year (blue), 3 years (purple), 5 years (orange). The x-axis indicates 1–specificity, and the y-axis represents sensitivity.

The model demonstrated favorable discriminative ability in both the training and validation cohorts. In the training cohort, the C-index was 0.783 (95% CI: 0.729–0.837) with an Akaike Information Criterion (AIC) value of 553.980. In the validation cohort, the C-index was 0.701 (95% CI: 0.605–0.797) with an AIC value of 310.943. As shown in **Figure 3**, receiver operating characteristic (ROC) analysis revealed that in the training cohort, the AUCs for predicting 1, 3, and 5 year OS were 0.814, 0.895, and 0.825, respectively. In the validation cohort, the corresponding AUCs were 0.778, 0.679, and 0.754.

**Figure 4:**
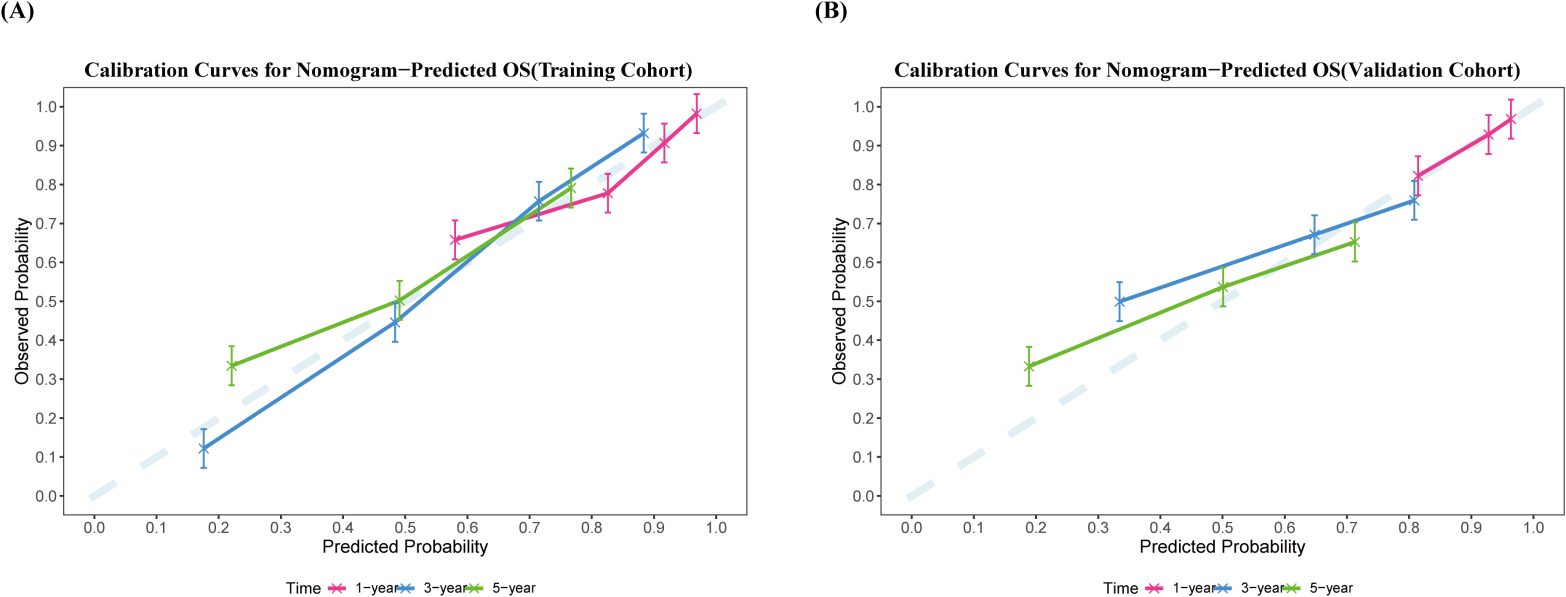
Calibration curves for the nomogram model predicting postoperative survival following HIFU treatment for HCC. **(A)** Training cohort; **(B)** validation cohort. The blue dashed line indicates the ideal calibration reference. The red, blue, and green solid lines represent 1,3 and 5 years postoperative OS prediction, respectively. The x-axis represents the event probability predicted by the nomogram, while the y-axis denotes the observed event probability.

Moreover, calibration curves based on 1000 bootstrap resampling iterations (**Figure 4**) indicated good agreement between the nomogram-predicted survival probabilities and the observed survival rates in both the training and validation cohorts. As summarized in **Table 3**, in the training cohort, the mean predicted survival probabilities at 1, 3, and 5 years after surgery were 0.835, 0.587, and 0.40, respectively, while the corresponding observed survival rates were 0.843, 0.586, and 0.551. The mean absolute errors (MAE) between predicted and observed values were 0.049, 0.049, and 0.031, respectively. Similarly, in the validation cohort, the mean predicted survival probabilities at 1, 3, and 5 years were 0.844, 0.604, and 0.422, with corresponding observed survival rates of 0.912, 0.652, and 0.543. The MAEs between predicted and observed values were 0.068, 0.141, and 0.150, respectively, indicating that the predictive performance of the nomogram remained relatively stable and reasonable in the internal validation.

**Table 3:** Calibration metrics of the nomogram model in the training and validation cohorts at different time points.

	Training cohort (n = 224)			Validation cohort (n = 163)		
Time	1 year	3 years	5 years	1 year	3 years	5 years
Mean Pred	0.835	0.587	0.401	0.844	0.604	0.422
Mean Act	0.843	0.586	0.551	0.912	0.652	0.543
MAE	0.050	0.049	0.031	0.068	0.141	0.150

**Figure 5:**
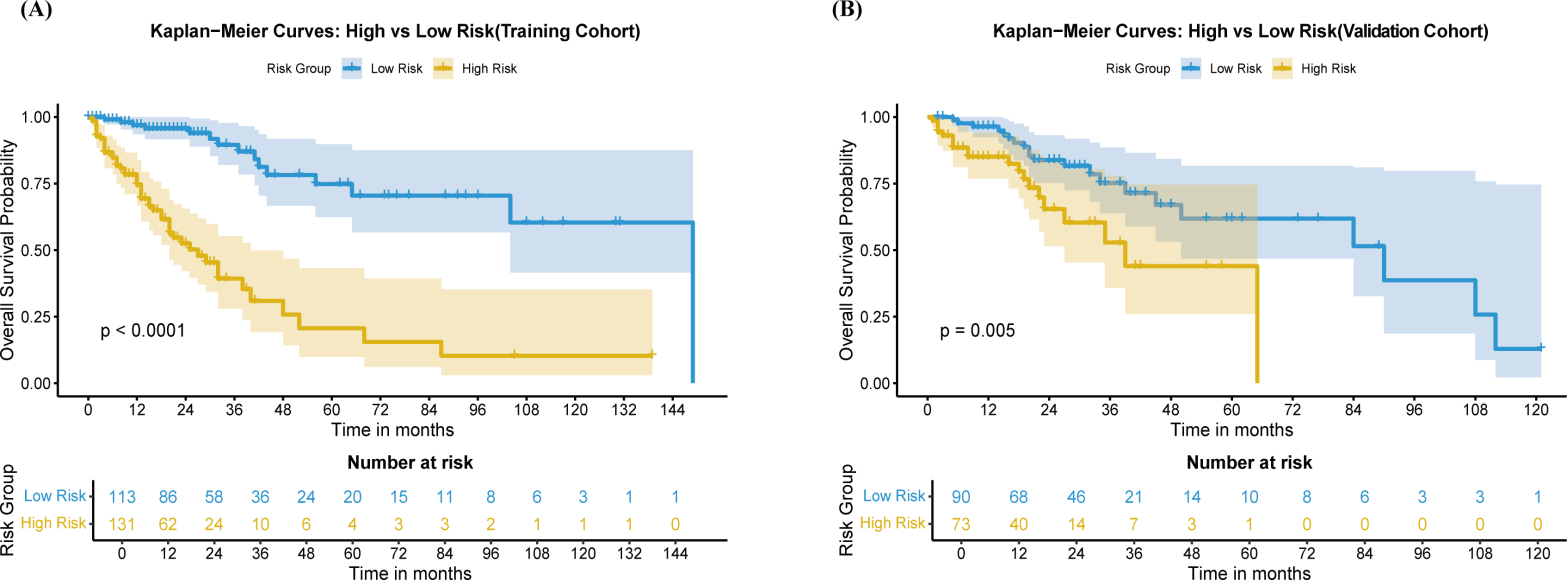
Kaplan-Meier curves for high-risk and low-risk groups following HCC HIFU treatment, based on nomogram model predictions. **(A)** Training Cohort **(B)** Validation Cohort. High risk (yellow), low risk (blue).

Based on the constructed nomogram model, an individualized risk score was calculated for each patient in the training cohort, aiming to convert the continuous risk predicted by the model into a discrete indicator suitable for clinical decision-making. Subsequently, using the ‘surv_cutpoint’ function (from the R ‘survminer’ package), all possible risk score values were iterated to identify the optimal cutoff value that maximized the difference in OS between high-risk and low-risk groups—i.e., the value yielding the maximum log-rank test statistic. Here and throughout, ‘NA’ denotes not available. In the training cohort, Kaplan-Meier analysis revealed a statistically significant difference in OS between the two groups (**Figure 5**). The Median Survival Time (MST) in the low-risk group was as long as 149 months (IQR: 104–NA), which was significantly better than the MST of 27 months (IQR: 20–40) in the high-risk group. Furthermore, the 1, 3, and 5 year OS rates in the low-risk group were 96.9%, 89.5%, and 74.8%, respectively, substantially higher than the corresponding rates of 74.6%, 39.3%, and 20.6% in the high-risk group.

This consistent stratification performance was further confirmed in the validation cohort. In the validation set, the low-risk group showed a favorable survival outcome, with an MST of 90 months (IQR: 50–NA) and 1-, 3-, and 5-year OS rates of 96.4%, 75.2%, and 61.8%, respectively. In contrast, the high-risk group had an MST of only 39 months (IQR: 27–NA) with corresponding OS rates of 85.1%, 52.8%, and 44.0% at the same time points. This pronounced risk-stratification capability provides a solid basis for individualized prognosis assessment and precise treatment decision-making in HCC, fully demonstrating the clinical guidance value of the model developed in this study.

**Figure 6:**
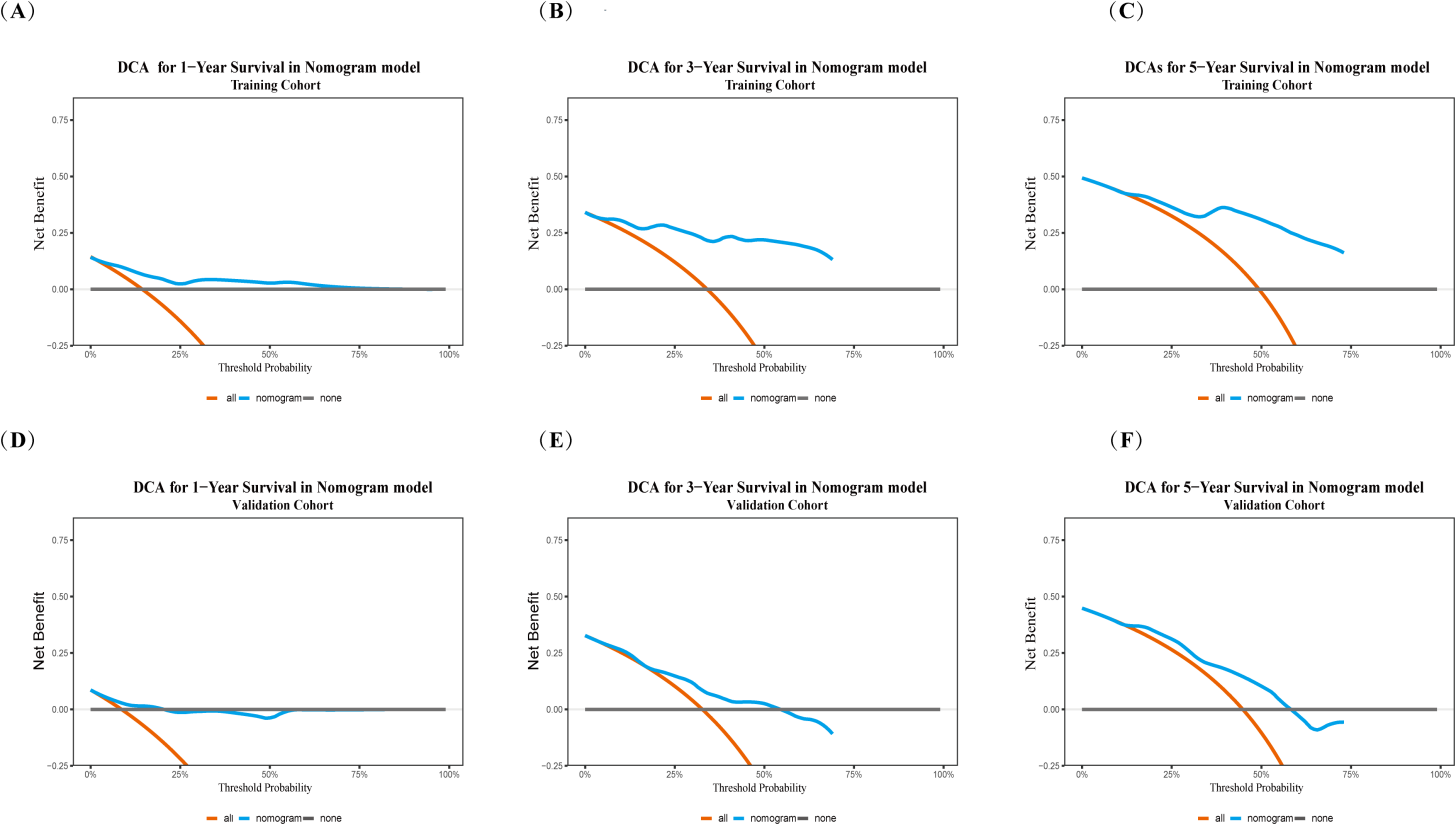
DCA curves of nomogram models predicting postoperative survival following HIFU treatment for HCC. Training Cohort: **(A)** 1-year postoperative OS; **(B)** 3-year postoperative OS; **(C)** 5-year postoperative OS; Validation Cohort: **(D)** 1-year postoperative OS; **(E)** 3-year postoperative OS; **(F)** 5-year postoperative OS. The x-axis represents the threshold probability, and the y-axis denotes net benefit. Treat all (orange), Treat none (gray), nomogram (blue).

DCA was further employed to evaluate the clinical net benefit of the nomogram. The decision curves (**Figure 6**) indicated that the nomogram provided clear clinical net benefit over a wide range of threshold probabilities. Specifically, in the training cohort, the net benefit threshold ranges for predicting 1-, 3-, and 5-year survival were 0–75%, 5–70%, and 15–75%, respectively. In the validation cohort, the corresponding net benefit threshold ranges were 5–20%, 20–60%, and 15–60% for 1-, 3-, and 5-year survival predictions.

### 3.4 AJCC TNM model

Based on the 8th edition AJCC staging system, HCC patients were classified into TNM stages through imaging and pathological confirmation. No significant difference in TNM stage distribution was observed between the training and validation cohorts (p = 0.527). Overall, the cohort comprised 83 patients with stage I, 99 with stage II, 23 with stage IIIA, 36 with stage IIIB, 27 with stage IVA, and 139 with stage IVB disease.

Following random allocation, the training cohort consisted of 43 stage I, 57 stage II, 15 stage IIIA, 19 stage IIIB, 19 stage IVA, and 91 stage IVB cases. Within this cohort, the MST for stages IIIA and IIIB was not reached during the follow-up period (i.e., over 50% of patients were still alive at the last follow-up). The median OS for patients with stage I, II, IVA, and IVB disease was 149, 68, 14, and 38 months, respectively. In the validation cohort, there were 40 stage I, 42 stage II, 8 stage IIIA, 17 stage IIIB, 8 stage IVA, and 48 stage IVB cases. The MST was not reached for stages I and IIIA. The median OS for patients with stage II, IIIB, IVA, and IVB disease was 108, 23, 35, and 65 months, respectively.

In the training cohort, the 8th edition AJCC staging system yielded a C-index of 0.673 (95% CI: 0.605–0.741) and an AIC of 597.43 for predicting survival after HIFU in HCC patients. In the validation cohort, the corresponding C-index was 0.649 (95% CI: 0.563–0.734) with an AIC of 319.73.

**Figure 7:**
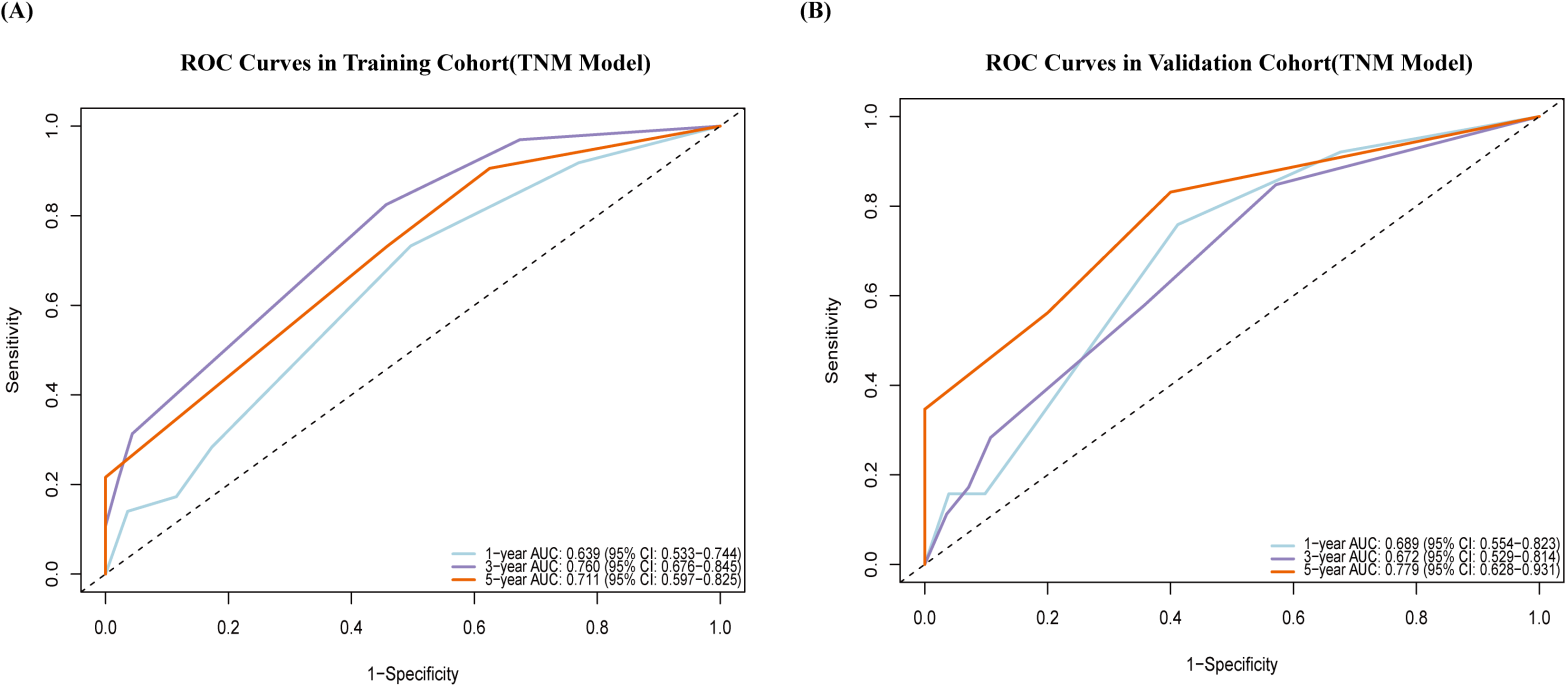
Time-dependent ROC curves for predicting postoperative survival rates in HCC following HIFU, based on the 8th edition AJCC staging system. **(A)** Training cohort; **(B)** validation cohort. AUC for predicting 1 year (blue), 3 years (purple), 5 years (orange). The x-axis indicates 1−specificity, and the y-axis represents sensitivity.

**Figure 7** presents the ROC analysis for risk prediction at various time points using the 8th edition AJCC staging system. In the training cohort, the AUCs (95% CI) for predicting 1, 3, and 5 year survival were 0.639 (0.533–0.744), 0.760 (0.676–0.845), and 0.711 (0.597–0.824), respectively. In the validation cohort, the corresponding AUCs were 0.689 (0.554–0.823), 0.672 (0.552–0.814), and 0.779 (0.628–0.937).

**Figure 8:**
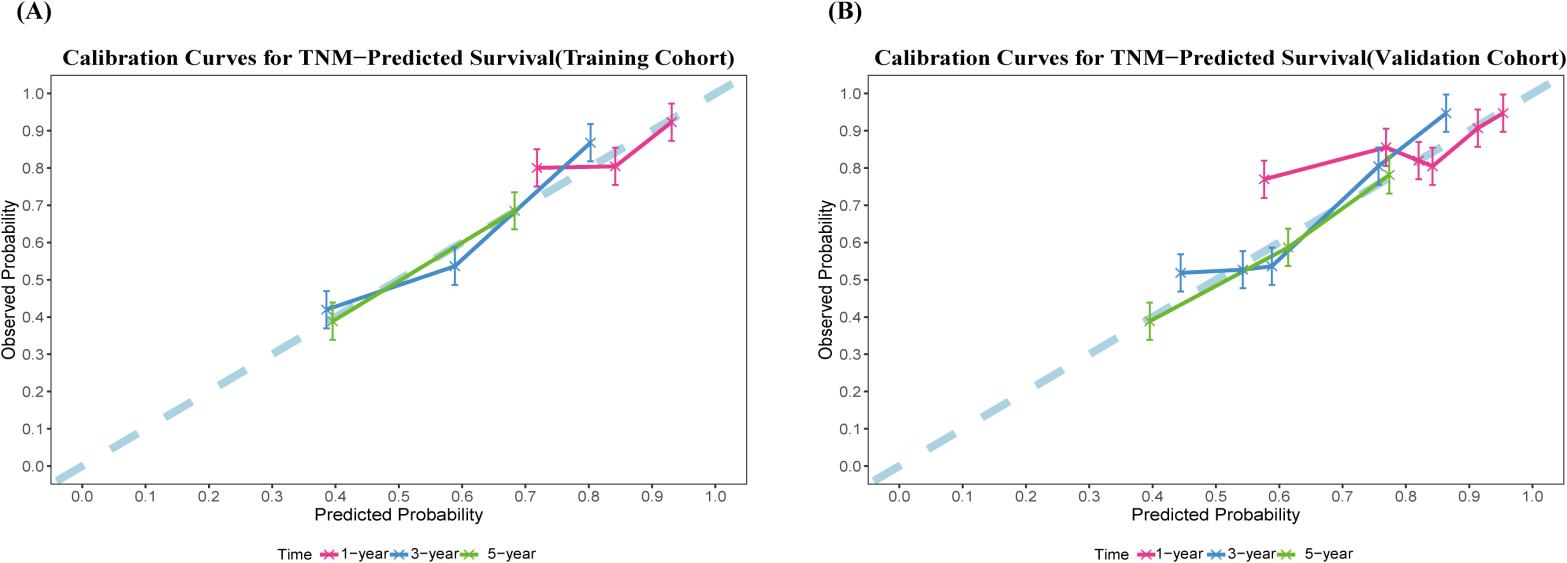
Calibration curves for the TNM model predicting postoperative survival following HIFU treatment for HCC. **(A)** Training cohort; **(B)** validation cohort. The blue dashed line indicates the ideal calibration reference. The red, blue, and green solid lines represent 1, 3 and 5 years postoperative OS prediction, respectively. The x-axis represents the event probability predicted by the TNM model, while the y-axis denotes the observed event probability.

Calibration of the 8th edition AJCC staging system, assessed via 1000 bootstrap resamples, varied across cohorts and time points (**Figure 8**). In the training cohort, the mean predicted survival probabilities at 1, 3, and 5 years were 0.853, 0.653, and 0.508, respectively, while the observed survival rates were 0.851, 0.633, and 0.458. The MAE values between predicted and observed values were 0.027, 0.138, and 0.177. In the validation cohort, the mean predicted probabilities were 0.863, 0.674, and 0.536, with corresponding observed rates of 0.914, 0.685, and 0.586. The MAE values were 0.050, 0.156, and 0.220, respectively(**Table 4**).

**Table 4:** Calibration metrics of the TNM model in the training and validation cohorts at different time points.

	Training cohort (n = 224)			Validation cohort (n = 163)		
Time	1 year	3 years	5 years	1 year	3 years	5 years
Mean pred	0.853	0.653	0.508	0.863	0.674	0.536
Mean act	0.851	0.633	0.458	0.914	0.685	0.586
MAE	0.027	0.138	0.177	0.050	0.156	0.220

**Figure 9:**
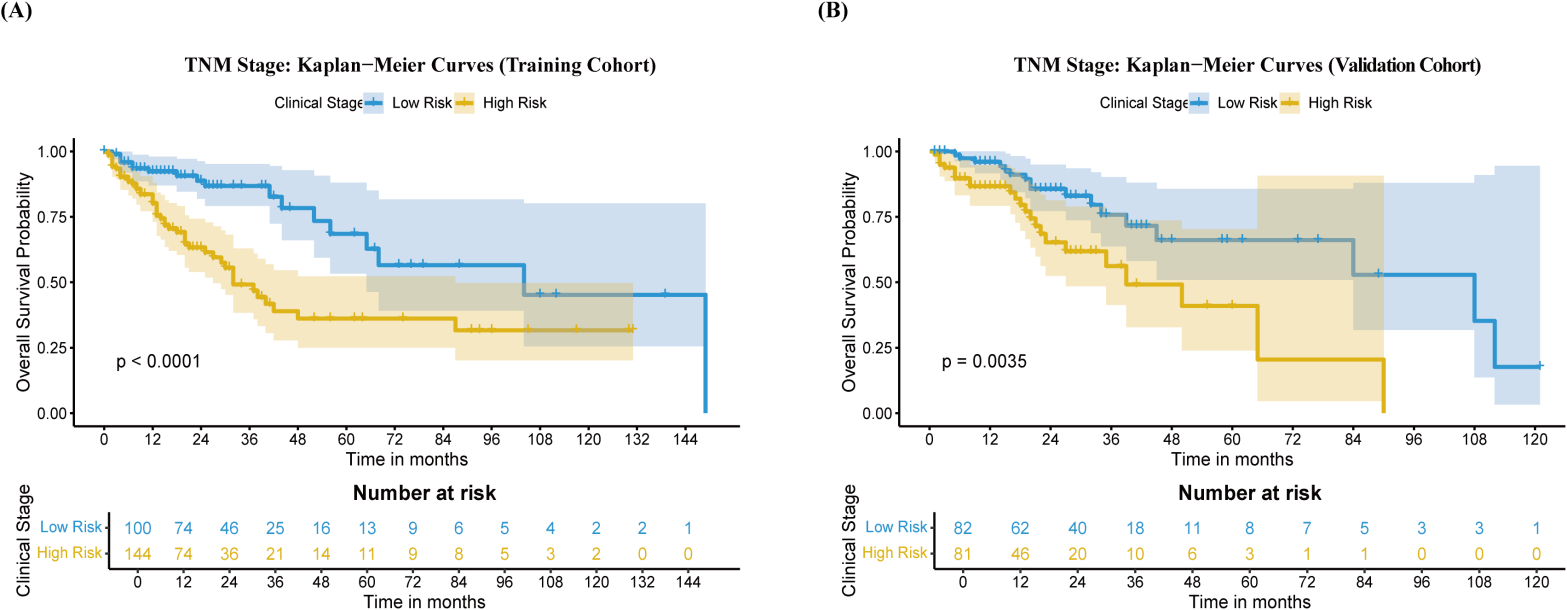
Kaplan-Meier curves for high-risk and low-risk groups following HCC HIFU treatment, based on the 8th edition AJCC TNM staging system in HCC patients after HIFU treatment. **(A)** Training Cohort **(B)** Validation Cohort. High risk (yellow), low risk (blue).

Based on the 8th edition AJCC TNM staging system, stages I/II were defined as the low-risk (early-stage) group and stages III/IV as the high-risk (advanced-stage) group. In the training cohort, the survival curves of the two groups were clearly separated (**Figure 9**). The MST in the low-risk group was 104 months (IQR: 65–NA), superior to the 32 months (IQR: 27–87) in the high-risk group. The 1-, 3-, and 5-year OS rates in the low-risk group were 92.3%, 86.8%, and 68.5%, respectively, compared with 80.2%, 49.1%, and 36.2% in the high-risk group.

In the validation cohort, the TNM staging system also demonstrated significant risk stratification capability (**Figure 9**). The MST was 108 months (IQR: 84–NA) in the low-risk group, compared with 39 months (IQR: 27–NA) in the high-risk group. Survival analysis revealed that the 1-, 3-, and 5-year OS rates in the low-risk group (96.0%, 75.8%, and 66.1%) were consistently and significantly higher than those in the high-risk group (86.7%, 56.2%, and 41.0%). Kaplan-Meier analysis confirmed that the stratification into high- and low-risk groups based on TNM stage was statistically significant in both cohorts.

**Figure 10:**
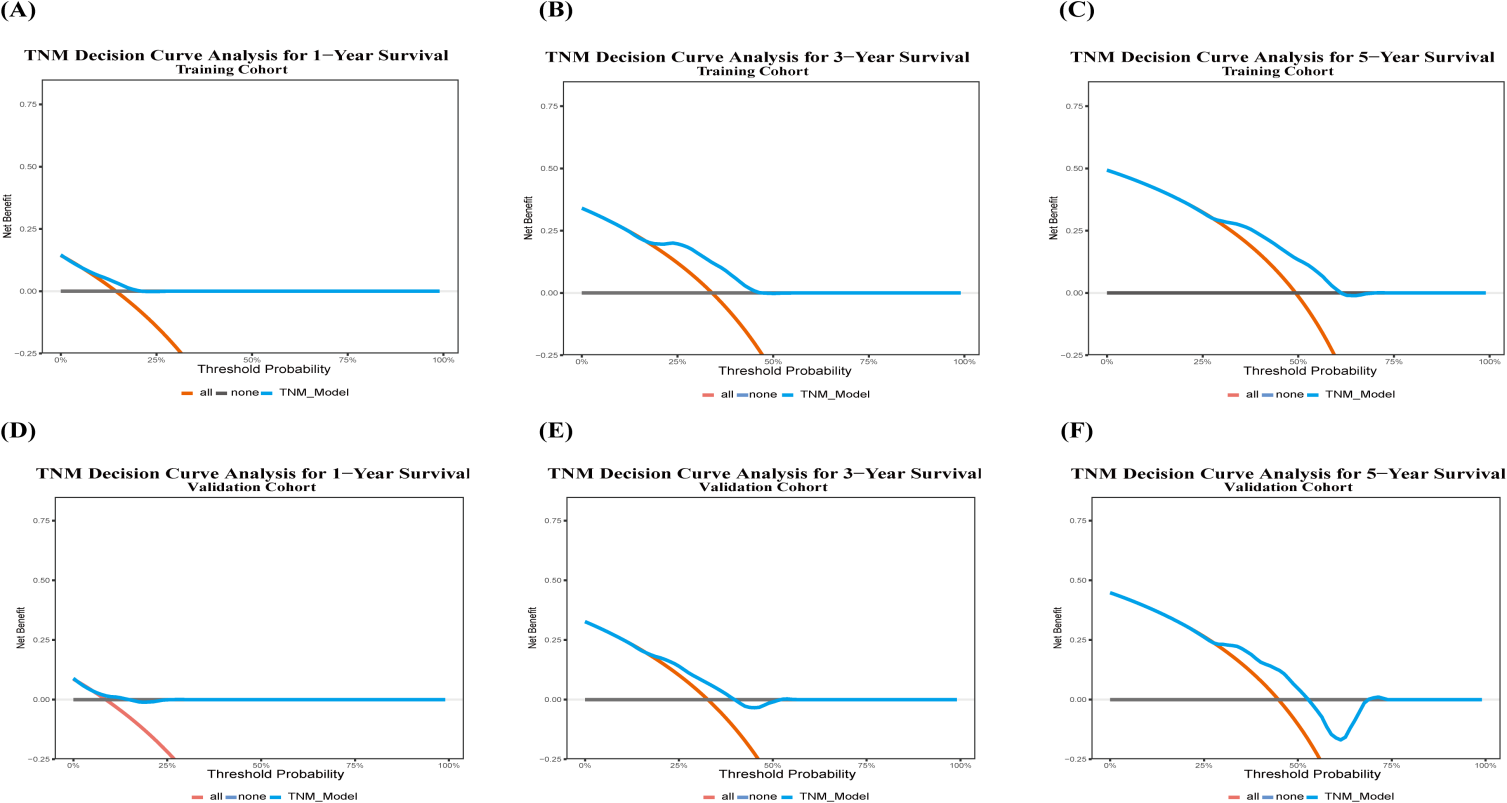
DCA of TNM models predicting postoperative survival following HIFU treatment for HCC. Training Cohort: **(A)** 1-year postoperative OS; **(B)** 3-year postoperative OS; **(C)** 5-year postoperative OS; Validation Cohort: **(D)** 1-year postoperative OS; **(E)** 3-year postoperative OS; **(F)** 5-year postoperative OS. The x-axis represents the threshold probability, and the y-axis denotes net benefit. Treat all (orange), Treat none (gray), nomogram (blue).

DCA (**Figure 10**) indicated that the clinical net benefit of the 8th edition AJCC staging system was relatively limited. In the training cohort, the threshold probability ranges yielding net benefit for predicting 1-, 3-, and 5-year survival were 10–20%, 20–50%, and 30–60%, respectively. In the validation cohort, the model showed no net benefit for predicting 1-year survival, while the net benefit ranges for 3- and 5-year survival were 20–40% and 30–55%, respectively.

### 3.5 Model comparison

The nomogram model developed in this study demonstrated favorable predictive and discriminatory performance in both the training and validation cohorts (**Table 5**). In the training cohort, the model achieved a C-index of 0.783, while in the validation cohort, the C-index reached 0.701. In contrast, the 8th edition AJCC TNM staging system performed poorly in predicting overall survival after HIFU treatment for HCC patients, with C-index values of only 0.673 and 0.649 in the training and validation cohorts, respectively.

**Table 5:** Comparison of the nomogram model with the 8th edition AJCC TNM model.

Evaluation indicators	Nomogram model						TNM model					
	Training cohort			Validation cohort			Training cohort			Validation cohort		
	1 year	3 years	5 years	1 year	3 years	5 years	1 year	3 years	5 years	1 year	3 years	5 years
C-index	0.780			0.700			0.673			0.649		
AIC	553.980			310.943			597.43			319.73		
AUC	0.814	0.895	0.825	0.778	0.679	0.754	0.639	0.760	0.711	0.689	0.672	0.779
Mean pred	0.835	0.587	0.401	0.844	0.604	0.422	0.853	0.653	0.508	0.863	0.674	0.536
Mean act	0.843	0.586	0.551	0.912	0.652	0.543	0.851	0.633	0.458	0.914	0.685	0.586
MAE	0.050	0.049	0.031	0.068	0.141	0.150	0.027	0.138	0.177	0.050	0.156	0.220
Net-benefit(%)	0-75	5-70	15-75	5-20	20-60	15-60	10-20	20-50	30-60	–	20-40	30-55

The agreement between predicted and observed survival rates was evaluated using calibration curves and MAE. Results indicated that the Nomogram exhibited good calibration in both cohorts. In the training cohort, predicted values closely matched the actual observations at all time points (12, 36, and 60 months), with MAE consistently maintained at very low levels (range: 0.031–0.050). In the validation cohort, the Nomogram retained stable predictive performance, with an MAE of 0.150 at the 60-month follow-up point, which remained within an acceptable range.

In comparison, the calibration performance of the TNM staging system deteriorated markedly over time. Although the TNM system showed reasonable accuracy in short-term prediction at 12 months (validation cohort MAE: 0.050), its predictive deviation increased substantially by 60 months, with MAE rising to 0.220 in the validation cohort. This indicates that the Nomogram offers higher consistency and reliability in individualized survival prediction, particularly in long-term survival assessment, compared to the traditional TNM staging system.

**Figure 11:**
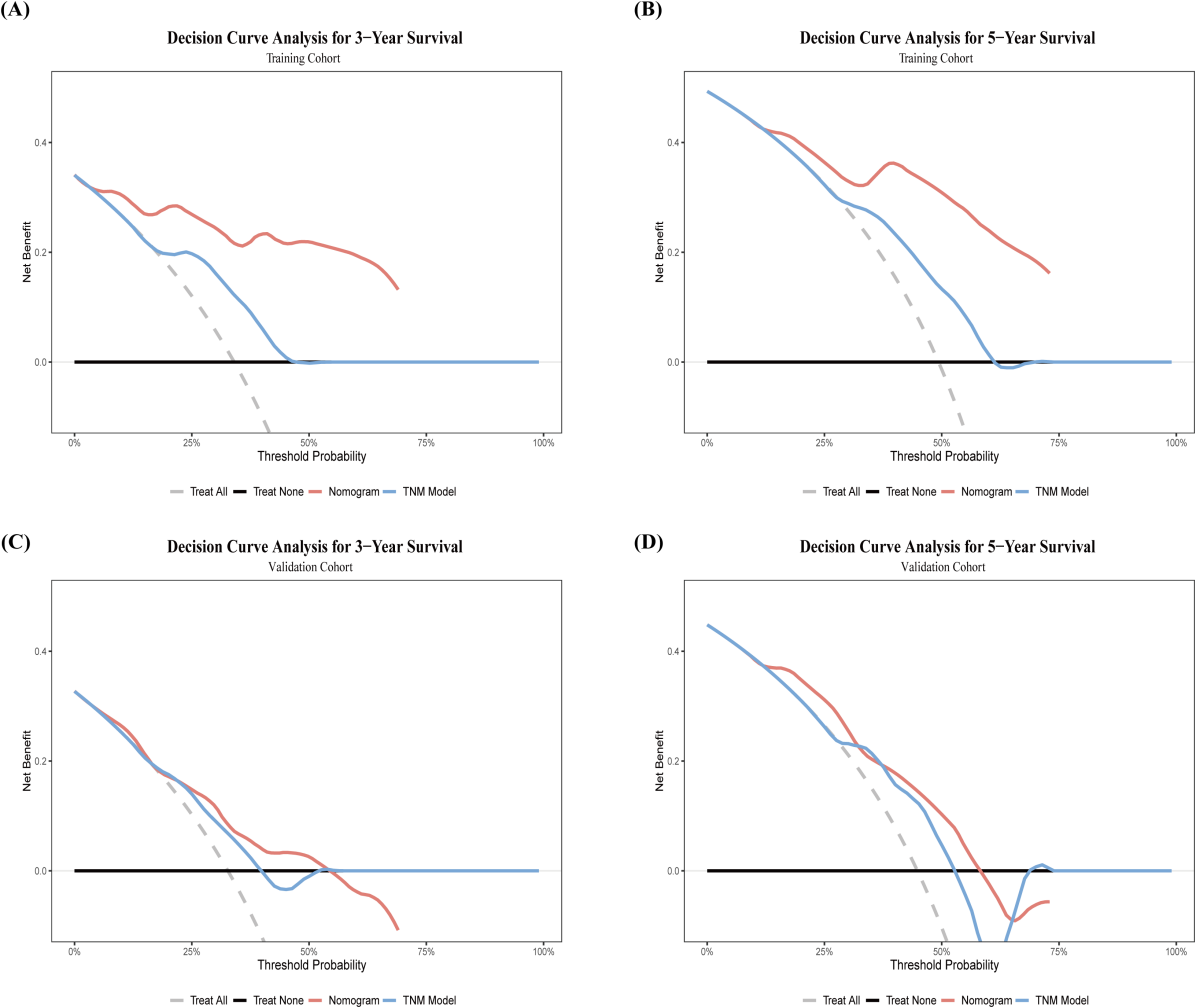
Comparison of DCA curves for predicting postoperative OS following HCC HIFU treatment. training cohort: **(A)** 3-year postoperative OS; **(B)** 5-year postoperative OS; Validation Cohort: **(C)** 3-year postoperative OS; **(D)** 5-year postoperative OS. Nomogram model (red). TNM staging system (blue). The x-axis represents the threshold probability, and the y-axis denotes net benefit. Treat all (gray dashed line), Treat none (black solid line).

DCA (**Figure 11**) demonstrated that the clinical model provided greater clinical net benefit and utility than the 8th edition AJCC TNM staging system in predicting 3- and 5-year survival rates. In the training cohort, both models showed net benefit within threshold probability ranges of 10–50% for 3-year survival and 20–63% for 5-year survival, with the clinical model yielding higher net gains. This advantage was confirmed in the validation cohort, where the clinical model maintained higher net benefit over broader threshold ranges (3-year: 30–55%; 5-year: 30–65%).

In summary, the nomogram model demonstrated significant superiority over the TNM staging system across multiple key metrics (**Table 5**). With respect to discriminative ability, calibration, and clinical utility, the Nomogram showed higher accuracy, robustness, and quantitatively interpretable results, along with a wider range of clinical net benefit. Therefore, the Nomogram model constructed in this study serves as a valuable complement to the conventional TNM staging system, offering a more precise and reliable tool for individualized prognostic evaluation in HCC patients following HIFU treatment.

## 4 Discussions

HCC is one of the most common malignancies in China. Extensive research has confirmed the efficacy of HIFU, either alone or in combination with interventional or systemic therapies, in the treatment of HCC. However, long-term survival outcomes following HIFU exhibit considerable heterogeneity (8, 9), and clinically applicable prognostic prediction models specifically for HIFU-treated patients are lacking. Therefore, there is an urgent need to establish a predictive model tailored to HIFU-treated HCC patients to assist in clinical decision-making. Although various algorithms, including machine learning, may offer potential advantages in predictive performance, this study ultimately opted to develop a nomogram model. Nomograms (17, 18)can translate the results of multivariate analysis into an intuitive graphical scoring system, thereby providing clinicians with an individualized and quantitative risk prediction tool.

Based on data from 407 HCC patients treated with HIFU, this study successfully developed and validated a clinical nomogram model for predicting OS. Univariate and multivariate Cox regression analyses identified maximum tumor diameter, number of tumor lesions, preoperative AFP, portal vein invasion, and preoperative LYMPH as independent risk factors affecting OS after HIFU treatment.

Maximum tumor diameter, number of tumor lesions, portal vein invasion, and AFP are well-established indicators of tumor aggressiveness (25, 26). Maximum tumor diameter and multifocality represent tumor burden; larger size and multiple lesions directly increase the risk of metastasis and complicate treatment. Large tumors pose technical challenges for complete ablation due to the heat sink effect from adjacent vessels and increased intratumoral heterogeneity (27). Multiple tumors suggest intrahepatic metastasis or multicentric occurrence, reflecting aggressive tumor biology and adding to therapeutic difficulty. Portal vein invasion (28) is a primary pathway for intrahepatic tumor dissemination and is strongly associated with a poor prognosis. As a diagnostic marker for HCC, elevated AFP correlates with higher pathological grades (29), indicating poor differentiation, active proliferation, and enhanced angiogenesis. High AFP is associated with unfavorable outcomes. These findings support the established principle that early intervention for small, solitary tumors yields the best prognosis and underscore the importance of careful patient selection for HIFU therapy.

In addition to validating traditional prognostic indicators that have been extensively confirmed in previous studies—such as maximum tumor diameter, number of tumor lesions, preoperative AFP, and portal vein invasion—our constructed nomogram model further identified preoperative LYMPH as an independent predictive factor. In prognostic studies of HCC, systemic immune-inflammatory markers, particularly the neutrophil-to-lymphocyte ratio (NLR) and platelet-to-lymphocyte ratio (PLR), have been widely recognized as robust prognostic predictors (30–33). These ratios collectively reflect the balance between pro-cancer inflammation and anti-tumor immunity. Compared with these composite ratios, our findings indicate for the first time that LYMPH alone possesses significant independent prognostic value, even without being combined into a ratio with other inflammatory markers. As a protective factor, preoperative LYMPH directly reflects the patient’s systemic immune status, highlighting the crucial role of host immunity in determining prognosis after HIFU ablation (34–36). This result aligns with the emerging concept of cancer immunoediting, in which lymphocytes mediate tumor surveillance and eliminate residual malignant cells after treatment (30, 37). Lymphopenia is associated with impaired anti-tumor immunity and increased risk of recurrence in various cancer types (31, 38). Protecting or enhancing lymphocyte function may represent a feasible strategy to improve HIFU outcomes, including the combined use of immunomodulators or the optimization of treatment timing to reduce immunosuppression. Lymphocytes are central effectors of anti-tumor immune responses, and a decrease in their number often indicates weakened immune surveillance and enhanced tumor immune escape.

The inclusion of LYMPH as a protective factor in the model introduces an immunological dimension to prognostic assessment. This finding corroborates the study by Rocco (39) et al. on the impact of systemic inflammatory markers on prognosis, underscoring the significance of host immune status in evaluating HIFU treatment efficacy. Compared with Xiong’s pathological model (40), the model proposed by Huang (21) et al. for predicting RFA prognosis, and the elderly-specific model by Tang (41) et al., our model incorporates an assessment of host immune status alongside conventional tumor burden and biological indicators. This design approach, which combines tumor characteristics with immune markers, reflects the current prognostic research emphasis on ‘host-tumor interactions’ and may contribute to the model’s favorable discriminatory ability. The establishment of this model not only provides a practical tool for prognostic evaluation after HIFU treatment, but the inclusion of immune markers also offers a reference for further exploration of the immunomodulatory mechanisms of HIFU therapy.

We integrated these factors into a visually interpretable nomogram model. The model demonstrated a C-index of 0.783 in the training cohort and 0.701 in the validation cohort, indicating good and stable discriminatory ability. Calibration curves confirmed the high accuracy of the nomogram predictions. In the training cohort, the mean predicted values closely matched the actual observations, with MAE at all time points remaining within a very low range of 0.031 to 0.050, fully supporting the model’s robustness and reliability across different time horizons. Similarly, the validation cohort showed good agreement, with MAE values below 0.15 at all time points, reflecting the model’s strong generalization ability. DCA further demonstrated that the model provides a net clinical benefit across a wide range of threshold probabilities, confirming its practical value in individualized treatment planning.

To evaluate its relative performance, our model was compared with previously published prognostic models for liver resection or other local ablation therapies. Its performance was comparable to or better than that of the model by Wang (33) et al. (training C-index = 0.79, validation C-index = 0.77) and the model by Zhang (42) et al. (training C-index = 0.64, validation C-index = 0.69). More importantly, this is the first prognostic tool specifically designed for HIFU-treated patients, offering unique clinical applicability. Furthermore, compared with the TNM staging system, our model provides more refined individual risk stratification, demonstrating that a multifactor-based integrated model outperforms traditional anatomical staging in the context of contemporary precision medicine.

This study has certain limitations. First, as a retrospective analysis, potential selection bias and uncontrolled confounding factors cannot be excluded. Second, although the model was validated internally, its generalizability requires further confirmation through multicenter prospective external cohorts. Third, given the pre-treatment clinical application of our nomogram, post-treatment variables such as immediate treatment success or subsequent adjuvant therapies were not incorporated into the primary model. Their potential impact on survival should be examined in future studies with standardized data collection. Fourth, detailed HIFU treatment parameters, including exposure time per spot, total delivered energy, and transducer element, were not included in the present analysis, which limits the ability to analyze their potential influence on prognosis. Moreover, the current median follow-up of approximately 32 months, while adequate for assessing short- to medium-term outcomes, remains insufficient for analyzing long-term recurrence and survival trends. An extended follow-up in the future will help to more comprehensively evaluate prognosis.

Building on the above discussion, future research could be conducted in the following directions: conducting multicenter prospective studies to further validate and refine the risk-stratification capability of the nomogram; incorporating dynamic indicators (such as changes in post-treatment LYMPH and AFP response) to enhance the timeliness and accuracy of the prediction model; exploring the integration of radiomic or molecular biomarkers to enable more personalized prognosis assessment; prospectively and systematically collecting detailed HIFU treatment parameters (e.g., exposure time, transducer element, energy) to investigate their prognostic value; and, importantly, designing clinical trials based on the risk strata defined by the model to compare the efficacy of HIFU alone versus HIFU combined with interventional therapies, thereby translating prognostic information into clinical treatment strategies.

## 5 Conclusions

In summary, this study successfully developed and validated a prognostic nomogram based on five readily accessible clinical variables to predict OS in HCC patients following HIFU therapy. The model demonstrated favorable discriminatory ability, with C-indices of 0.783 in the training cohort and 0.701 in the validation cohort, and its predictive performance surpassed that of the conventional TNM staging system. By incorporating preoperative LYMPH, maximum tumor diameter, AFP, number of tumor lesions, and portal vein invasion, this nomogram provides a practical tool for individualized assessment of 1, 3, and 5 years survival probabilities after HIFU ablation.

## Data Availability

The raw data supporting the conclusions of this article will be made available by the authors, without undue reservation.
